# IGF-I, IGF-II, and Insulin Stimulate Different Gene Expression Responses through Binding to the IGF-I Receptor

**DOI:** 10.3389/fendo.2013.00098

**Published:** 2013-08-09

**Authors:** Soetkin Versteyhe, Birgit Klaproth, Rehannah Borup, Jane Palsgaard, Maja Jensen, Steven G. Gray, Pierre De Meyts

**Affiliations:** ^1^Receptor Systems Biology Laboratory, Hagedorn Research Institute, Novo Nordisk A/S, Gentofte, Denmark; ^2^Genomic Medicine, Microarray Center, Copenhagen University Hospital, Copenhagen, Denmark; ^3^Thoracic Oncology Research Group, Trinity Centre for Health Sciences, Institute of Molecular Medicine, St. James’s Hospital, Dublin, Ireland

**Keywords:** IGF-I receptor, microarray gene expression, insulin, IGF, differential signaling

## Abstract

Insulin and the insulin-like growth factors (IGF)-I and -II are closely related peptides important for regulation of metabolism, growth, differentiation, and development. The IGFs exert their main effects through the IGF-I receptor. Although the insulin receptor is the main physiological receptor for insulin, this peptide hormone can also bind at higher concentrations to the IGF-I receptor and exert effects through it. We used microarray gene expression profiling to investigate the gene expression regulated by IGF-I, IGF-II, and insulin after stimulation of the IGF-I receptor. Fibroblasts from mice, knockout for IGF-II and the IGF-II/cation-independent mannose-6-phosphate receptor, and expressing functional IGF-I but no insulin receptors, were stimulated for 4 h with equipotent saturating concentrations of insulin, IGF-I, and IGF-II. Each ligand specifically regulated a group of transcripts that was not regulated by the other two ligands. Many of the functions and pathways these regulated genes were involved in, were consistent with the known biological effects of these ligands. The differences in gene expression might therefore account for some of the different biological effects of insulin, IGF-I, and IGF-II. This work adds to the evidence that not only the affinity of a ligand determines its biological response, but also its nature, even through the same receptor.

## Introduction

Insulin and the closely related insulin-like growth factors (IGF)-I and -II are important for the regulation of metabolism and cell growth, survival, motility, differentiation, and development (1–6). These ligands bind to closely related receptor tyrosine kinases. The main physiological receptor for insulin is the insulin receptor, while the IGFs mainly exert their effects through the IGF-I receptor (7, 8). The insulin receptor exists under two isoforms, A and B, due to alternative splicing of exon 11 of the insulin receptor gene (9, 10).

Insulin-like growth factor-II in mammals also binds to the IGF-II/cation-independent mannose-6-phosphate receptor, which is thought to act as a scavenger for IGF-II rather than a signaling receptor (11, 12). Its presence on most cells however complicates the study of IGF-II binding and signaling mediated through the IGF-I receptor.

Binding of the ligands to the insulin or IGF-I receptor leads to autophosphorylation of the receptor on tyrosine residues. This creates binding sites for SH2 and PTB domain-containing docking proteins such as IRS-1–4 and Shc, and stimulates the tyrosine kinase activity of the receptor, enabling it to phosphorylate multiple cytoplasmic substrates, which activates signaling cascades, resulting in ligand-specific biological effects (4, 13).

Both the ligands and the receptors are closely related (and therefore the ligands can bind to their non-cognate receptors) and the signaling pathways they activate are largely overlapping (14). Microarray profiling showed that the two receptors are capable of stimulating the same gene expression response (15). Nevertheless, insulin is mainly a metabolic regulator, while the IGFs exert mainly mitogenic effects (growth, proliferation …). The molecular basis of this signaling specificity is still not understood (6, 16).

As mentioned, the three ligands can also bind to their non-cognate receptors, though with lower affinity, and by doing so they can exert different effects in comparison to the cognate ligand. Frasca et al. and Morrione et al., e.g., showed independently that IGF-II is more potent in stimulating proliferation through the insulin receptor A isoform than insulin (17, 18). Frasca et al. also showed that insulin is a more potent metabolic regulator through this isoform than IGF-II (17). Pandini et al. found that insulin and IGF-II induce different gene expression patterns after binding to the A isoform of the insulin receptor (19). Malaguarnera et al. found that proinsulin binds with high affinity the insulin receptor isoform A and predominantly activates the mitogenic pathway (20). Also, insulin analogs with different residence times on the insulin receptor have been shown to have different relative potencies for mitogenic versus metabolic signaling (21–23). Previous work from our laboratory has described an insulin mimetic peptide that despite binding to the insulin receptor with an affinity similar to insulin’s is less potent in stimulating thymidine incorporation and induces a different gene expression response in comparison to insulin (24). All in all, it is becoming increasingly clear that various ligands acting through the same receptor may activate different patterns of end-point cellular effects (“differential signaling”).

In this study we measured gene expression by microarray profiling after stimulating mouse fibroblasts expressing the IGF-I receptor, but devoid of insulin and IGF-II/cation-independent mannose-6-phosphate receptors (25) with equipotent concentrations of insulin, IGF-I, and IGF-II. During the analysis the focus was on finding differences, rather than similarities, in gene expression between the three ligands. The results show that insulin, IGF-I, and IGF-II indeed create different gene expression responses when stimulating the IGF-I receptor. We hope that these results and further studies will lead to a better understanding of the signaling specificity and different biological effects of these three ligands.

## Materials and Methods

### Materials

Fibroblasts from mice knockout for IGF-II and the IGF-II/cation-independent mannose-6-phosphate receptor were a gift from Dr. Kurt von Figura (25). Insulin was from Novo Nordisk A/S, Denmark, and IGF-I and IGF-II from Novozymes GroPep, Thebarton, SA, Australia. ^125^I-IGF-I was prepared by Novo Nordisk A/S. Unless otherwise specified all chemicals were from Sigma-Aldrich, Denmark.

### Cell line and culture conditions

The mouse fibroblasts were routinely cultured in 80 cm^2^ TC flasks (Nunc, Denmark) in DMEM medium (with Glutamax-1 and 4.5 g/l glucose; Gibco, Invitrogen, Denmark) supplemented with 10% Fetal Bovine Serum (Gibco, Invitrogen, Denmark), 100 U/ml Penicillin, and 100 μg/ml Streptomycin (Gibco, Invitrogen, Denmark). The cells were grown at 37°C in a 5% CO_2_ humidified atmosphere. They were passaged three times a week by washing in D-PBS (w/o Calcium and Magnesium; Gibco, Invitrogen, Denmark), trypsinization in Trypsin-EDTA (Gibco, Invitrogen, Denmark), and subsequent resuspension and dilution in fresh medium.

The mouse fibroblasts, devoid of IGF-II and the IGF-II/cation-independent mannose-6-phophate receptor, did not bind ^125^I-insulin, indicating the absence of biologically active insulin receptors (results not shown), but did bind ^125^I-IGF-I. From the below mentioned homologous competition assay data, we found that approximately 75,000 IGF-I receptor sites/cell are present on this cell line.

### Determining the affinities of IGF-I, IGF-II, and insulin for the IGF-I receptor

To determine the apparent affinities of the ligands for the IGF-I receptor on the mouse fibroblast cell line, homologous and heterologous radioligand competition assays were performed in quadruplets. Cells were detached with 10 mM EDTA (Gibco, Invitrogen, Denmark). Three million cells per milliliter were incubated with a constant concentration of ^125^I-IGF-I (20,000 cpm/ml) and increasing concentrations of cold IGF-I, IGF-II, or insulin for 2.5 h (time needed to reach steady-state binding) at 15°C in Hepes Binding Buffer (100 mM Hepes, 120 mM NaCl, 5 mM KCl, 1.2 mM MgSO_4_, 1 mM EDTA, 10 mM Glucose, 15 mM Na Acetate, and 1% BSA). After centrifugation unbound ^125^I-IGF-I was removed and cell-bound ^125^I-IGF-I was counted in a Wallac WIZARD gamma counter (PerkinElmer). *K*_d_ values were calculated after fitting the data to a one-site model using a program developed in our laboratory by Ronald M. Shymko and Andreas V. Groth.

### Preparation of the cells for the microarray experiments

Mouse fibroblasts were seeded out into 145 cm^2^ TC dishes (Nunc, Denmark) at two million cells per dish and subsequently allowed to recover for 24 h. In quadruplets, but at the same cell passage and after washing the cells twice with D-PBS (w/o Calcium and Magnesium; Gibco, Invitrogen, Denmark), the cells were serum starved for 24 h and afterward either left unstimulated or stimulated for 4 h with 20 nM IGF-I, 177 nM IGF-II, or 5168 nM insulin. These concentrations compensate for the relative affinities of the ligands for the receptor, measured as described above.

### Isolation and purification of total RNA

Total RNA was isolated by using the TRI^®^ reagent method (Molecular Research Gene, USA) and cleaned up using the RNeasy™ Mini Kit (Qiagen) according to the manufacturers’ protocol. RNA quality was verified by 1% agarose gel electrophoresis. Concentration and purity were determined by measuring absorbance at *A*_260_ and *A*_280_ in a spectrophotometer (Brinkmann Eppendorf BioPhotometer, Germany).

### cRNA generation and hybridization to gene chip microarrays

cRNA was produced using the One-cycle Target Labeling Kit (Affymetrix, Santa Clara, CA, USA). One-cycle Target Labeling Kit and procedures followed protocols in the GeneChip Expression Analysis Technical Manual (Affymetrix, Santa Clara, CA, USA). Fragmented biotin-labeled cRNA was hybridized to Affymetrix GeneChip^®^ Mouse Genome 430 2.0 Arrays according to manufacturer’s protocol. The arrays were incubated at 45°C for 16 h under rotation (60 rpm), washed in the GeneChip^®^ Fluidics Station (Affymetrix) and scanned using the GeneChip^®^ Scanner 3000.

### Data analysis

The quality of the arrays was verified by quality control in the R package^1^ from Bioconductor^2^. The probe level data (CEL files) were transformed into expression values using R and the GC-RMA package from Bioconductor (see text footnote 2) (26). Briefly, the background was subtracted, the data were normalized by the quantile normalization method and the expression values of a probe set were summarized into one expression value.

For data analysis, the expression values were imported into the software package DNA-Chip Analyzer (dChip) (version 2008), freeware developed by Li and Wong (27)^3^. When generating original lists of transcripts, a fold change and *p*-value cut-off of respectively 1.2 and 0.05 were chosen. The lower confidence bound of fold changes was used for filtering and the threshold for absolute difference between two group means was set to 35. Using these cut-offs gave empirical median false discovery rates (FDR) of maximum 2% after running 100 permutations in dChip (FDRs were 0% for all but the lists of genes regulated by insulin, IGF-I, or IGF-II in comparison to the control). dChip recommends a median FDR of ¡5 or 10%. Composing a list of transcripts regulated by insulin, IGF-I, and IGF-II together or separately was done by selecting transcripts that fulfilled the above-mentioned criteria for the ligands in comparison to the control. In order to generate lists containing transcripts only regulated by one of the ligands, transcripts were selected that fulfilled the criteria for one of the ligands in comparison to the control and in comparison to the two other ligands. Transcripts that also fulfilled the criteria for one of the other ligands in comparison to the control were excluded. The resulting transcripts, uniquely regulated by one of the ligands, were afterward filtered for a fold change of 1.5 in comparison to the control, in order to focus the below mentioned functional analysis on the transcripts with the highest biological relevance. In order to study differences between one ligand and the two other ligands as a group, transcripts were selected that fulfilled the criteria for the two ligands in comparison to the control and to the other ligand. The resulting transcripts were afterward filtered for a fold change of 1.5 in comparison to the control, in order to focus the below mentioned functional analysis on the transcripts with the highest biological relevance.

Identification of gene function themes and canonical pathways was done using the web-based software Ingenuity Pathways Analysis (IPA)^4^. IPA takes the gene IDs in the dataset file and maps them to genes in the Ingenuity Pathways Knowledge Base (IPKB). The functional and canonical pathway analyses identified the molecular and cellular functions and canonical pathways that were most significant to the data set. This significance value is a measure for how likely it is that genes from the dataset file under investigation participate in that function. In this method, the *p*-value is calculated by comparing the number of user-specified genes of interest that participate in a given function or pathway, relative to the total number of occurrences of these genes in all functional/pathway annotations stored in the IPKB. Ingenuity uses a right-tailed Fisher’s Exact Test in order to calculate a *p*-value. In the right-tailed Fisher’s Exact Test, only over-represented functional/pathway annotations, annotations which have more Functions/Canonical Pathways Analysis Genes than expected by chance (“right-tailed” annotations), are used.

### Preparation of total RNA for qRT-PCR

To validate the microarray data two-step RT-PCR was performed on a subset of genes. To perform the validation on biological replicates, new (in comparison to the RNA used for the arrays) total RNA samples were prepared at three different cell passages.

### qRT-PCR

The total RNA was reverse transcribed into single-stranded cDNA using the Transcriptor First Strand cDNA Synthesis Kit (Roche Applied Science) according to the manufacturer’s protocol. The cDNA was transcribed using FastStart TaqMan Probe Master (Rox) (Roche Applied Science). Probes were purchased from Universal ProbeLibrary (Roche). Probes were selected and primer sequences designed using the ProbeFinder software (Universal ProbeLibrary, Roche). The primers were purchased from DNA-technologies, Denmark. Primers and probes used are listed in Table 1. Per qRT-PCR assay the cDNA samples were run in quadruplets with 18S as the internal control gene, in 384-well optical plates on an ABI 7900HT Prism sequence detection system (Applied Biosystems). Each 15 μl TaqMan reaction contained 1.5 μl cDNA, 7.5 μl 2× FastStart TaqMan Probe Master (Rox), 0.15 μl Universal Probe (10 μM), 0.15 μl left primer (20 μM), 0.15 μl right primer (20 μM), and 5.55 μl PCR-grade water. PCR parameters were 50°C for 2 min, 95°C for 10 min, 40 cycles of 95°C for 15 s, and 60°C for 1 min. For each gene and for each biological replicate TaqMan PCR assays were performed in triplicates. The data were analyzed using Sequence Detector Software (Applied Biosystems), where after the fold changes were calculated by use of the ΔΔ*C*_t_ method (28). To compare the qRT-PCR data with the microarray results, negative microarray fold changes were converted into values between 0 and 1. When multiple probe sets for one gene were regulated on the microarrays, the average fold change was calculated. Significant differences in the qRT-PCR data were calculated by a two-tailed *t*-test.

**Table 1:** **Primers and probes used for qRT-PCR**.

Transcript	Accession nr.	Universal probe no.	Primer	Sequence 5′–3′
18S		77	left	gattgatagctctttctcgattcc
			Right	gacaaatcgctccaccaact
Ccnd1 (cyclin D1)	NM_007631	67	Left	gagattgtgccatccatgc
			Right	ctcttcgcacttctgctcct
Areg (amphiregulin)	NM_009704	73	Left	gacaagaaaatgggactgtgc
			Right	ggcttggcaatgattcaact
Egr2 (early growth response 2)	X06746	60	Left	ctacccggtggaagacctc
	NM_010118		Right	aatgttgatcatgccatctcc
HB-EGF (heparin-binding EGF-like growth factor)	L07264	55	Left	cgtgggacttctcatgtttagg
	NM_010415		Right	cgcccaacttcactttctct
Dusp6 (dual specificity phosphatase 6)	NM_026268	66	Left	tggtggagagtcggtcct
			Right	tggaacttactgaagccacctt
Jun-B (Jun-B oncogene)	NM_008416	3	Left	accacggagggagagaaaag
			Right	agttggcagctgtgcgtaa

## Results

### Affinities of IGF-I, IGF-II, and insulin for the IGF-I receptor

**Figure 1:**
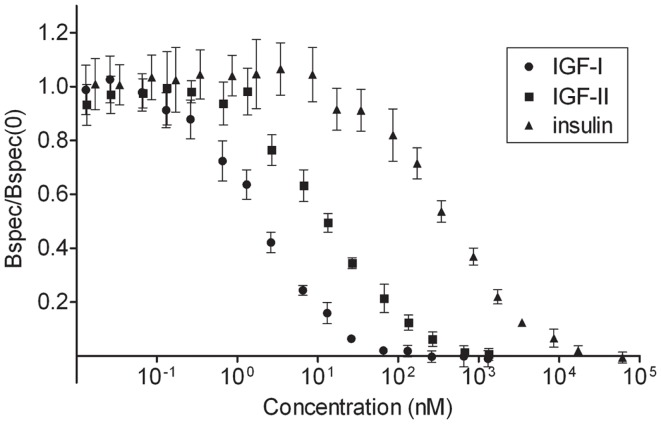
**Affinities of insulin, IGF-I, and IGF-II for the IGF-I receptor**. To determine the apparent affinities of the ligands for the IGF-I receptor on the mouse fibroblasts, homologous and heterologous radioligand competition assays were performed in quadruplets. Three million cells/ml were incubated with a constant concentration of ^125^I-IGF-I (20,000 cpm/ml) and increasing concentrations of cold IGF-I, IGF-II, or insulin for 2.5 h (time needed to reach steady-state binding) at 15°C. After centrifugation unbound ^125^I-IGF-I was removed and bound ^125^I-IGF-I was counted in a gamma counter. Specifically bound ^125^I-IGF-I/specifically bound ^125^I-IGF-I at 0 nM cold ligand was plotted versus the concentration of cold ligand. *K*_d_ values were calculated after fitting the data to a one-site model using a program developed by Ron M. Shymko and Andreas V. Groth. Results are averages ± standard deviations.

In order to stimulate the IGF-I receptor on mouse fibroblasts with concentrations that are adjusted for the relative affinities of IGF-I, IGF-II, and insulin for the receptor, the apparent affinities of the three ligands were measured by allowing the cold ligands to compete with ^125^I-IGF-I for binding to the IGF-I receptor (Figure 1). IGF-I had a *K*_d_ value of 1.49 ± 0.14 nM, IGF-II a *K*_d_ value of 13.11 ± 0.69 nM, and insulin of 383 ± 27 nM. These results are in accordance with the known relative affinities of the ligands for the IGF-I receptor (29). Taking these relative affinities into account, it was decided to stimulate the cells for 4 h with 20 nM IGF-I, 177 nM IGF-II, or 5168 nM insulin, concentrations then are near saturation of the receptor with either ligand.

### Global gene regulation patterns

A total of 698 transcripts were regulated by both insulin and the IGFs (fold changes and *p*-values for these transcripts are in Table S1 in Supplementary Material). Table 2 shows the number of transcripts regulated by each ligand in comparison to the control and the number of transcripts commonly regulated between ligands. Fold changes and *p*-values for these transcripts can be found in Table S2 in Supplementary Material (IGF-I), Table S3 in Supplementary Material (IGF-II), and Table S4 in Supplementary Material (insulin). All the transcripts regulated in common between ligands were either up-regulated by all regulating ligands or down-regulated by all regulating ligands. Even though the three ligands stimulate similar responses, the overlap is partial and we identified transcripts selectively regulated by each ligand.

**Table 2:** **Global gene regulation patterns**.

	Transcripts regulated in comparison to control	Fraction of transcripts also regulated by IGF-I	Fraction of transcripts also regulated by IGF-II	Fraction of transcripts also regulated by insulin
IGF-I	2715		1213	754
IGF-II	1779	1213		956
Insulin	1215	754	956	

*The number of transcripts regulated by each of the three ligands in comparison to the control and the number of transcripts commonly regulated between ligands (in comparison to the control) are shown. Cut-offs for fold change and p-value are 1.2 and 0.05 respectively*.

### Transcripts selectively regulated by IGF-I, IGF-II, or insulin

#### Transcripts selectively regulated by IGF-I

A total of 75 transcripts were only regulated by IGF-I (Table 3; fold change cut-off 1.5). Fold changes and *p*-values for insulin and IGF-II can be found in Table S5 in Supplementary Material.

**Table 3:** **Transcripts selectively regulated by IGF-I**.

Transcript	Probe set (Affymetrix)	Accession nr.	Fold change	*p*-Value
Eif5: eukaryotic translation initiation factor 5	1415723_at	BQ176989	1.54	0.000187
Srp54a /// Srp54b /// Srp54c: signal recognition particle 54a /// signal recognition particle 54b /// signal recognition particle 54C	1416153_at	NM_011899	1.55	0.003558
Pafah1b1: platelet-activating factor acetylhydrolase, isoform 1b, beta1 subunit	1417086_at	BE688382	1.69	0.005799
Dnaja2: DnaJ (Hsp40) homolog, subfamily A, member 2	1417182_at	C77509	1.67	0.000129
Orc2l: origin recognition complex, subunit 2-like (*S. cerevisiae*)	1418226_at	BB830976	1.77	0.000088
Ctcf: CCCTC-binding factor	1418330_at	BB836888	1.53	0.026056
AI837181: expressed sequence AI837181	1418775_at	NM_134149	−1.86	0.007512
Il17rc: interleukin 17 receptor C	1419671_a_at	NM_134159	−1.80	0.006468
Supt16h: suppressor of Ty 16 homolog (*S. cerevisiae*)	1419741_at	AW536705	1.52	0.002900
Nap1l1: nucleosome assembly protein 1-like 1	1420477_at	BG064031	1.51	0.000989
Shoc2: soc-2 (suppressor of clear) homolog (*C. elegans*)	1423129_at	BQ032685	1.51	0.000692
Lin7c: lin-7 homolog C (*C. elegans*)	1423322_at	BQ176612	1.68	0.000844
Stk17b: serine/threonine kinase 17b (apoptosis-inducing)	1423452_at	AV173139	1.64	0.000103
Usp1: ubiquitin specific peptidase 1	1423675_at	BC018179	1.55	0.008911
Nop14: NOP14 nucleolar protein homolog (yeast)	1423991_at	BC024998	1.75	0.001692
Uso1: USO1 homolog, vesicle docking protein (yeast)	1424274_at	BC016069	1.77	0.002483
Flad1: RFad1, flavin adenine dinucleotide synthetase, homolog (yeast)	1424421_at	BC006806	−1.59	0.004350
Rbm26: RNA binding motif protein 26	1426803_at	BM120471	1.71	0.031929
Ythdf3: YTH domain family 3	1426841_at	BB183208	1.68	0.014072
Rbbp8: retinoblastoma binding protein 8	1427061_at	BB167067	1.56	0.000050
Zc3h15: zinc finger CCCH-type containing 15	1427876_at	BB703070	1.65	0.000917
Zmpste24: zinc metallopeptidase, STE24 homolog (*S. cerevisiae*)	1427923_at	BM233793	1.52	0.005861
Spin4: spindlin family, member 4	1427985_at	BC027796	2.17	0.001115
Fip1l1: FIP1 like 1 (*S. cerevisiae*)	1428280_at	BM199874	1.59	0.022198
2810026P18Rik: RIKEN cDNA 2810026P18 gene	1428529_at	AK012825	1.57	0.016748
Uba6: ubiquitin-like modifier activating enzyme 6	1428945_at	BB417360	1.73	0.001773
Cep57: centrosomal protein 57	1428968_at	AW457682	1.58	0.006762
Nat13: *N*-acetyltransferase 13	1428970_at	AV113878	1.82	0.000018
1300003B13Rik: RIKEN cDNA 1300003B13 gene	1429690_at	AK004870	1.56	0.012148
9030419F21Rik: RIKEN cDNA 9030419F21 gene	1433101_at	AK018519	−1.70	0.026402
Ddx52: DEAD (Asp-Glu-Ala-Asp) box polypeptide 52	1434608_at	BB132474	1.71	0.001952
Ankle2: ankyrin repeat and LEM domain containing 2	1434721_at	AV378849	1.50	0.009946
Wapal: wings apart-like homolog (*Drosophila*)	1434835_at	BM230523	1.59	0.006908
Tsr2: TSR2, 20S rRNA accumulation, homolog (*S. cerevisiae*)	1435170_at	BQ177187	1.89	0.021023
Ube2n: ubiquitin-conjugating enzyme E2N	1435384_at	BE980685	1.79	0.000704
Trpm4: transient receptor potential cation channel, subfamily M, member 4	1435549_at	BI685685	−1.59	0.007237
Scyl2: SCY1-like 2 (*S. cerevisiae*)	1436313_at	BM249802	1.91	0.003117
Mmgt1: membrane magnesium transporter 1	1436705_at	BB262218	1.89	0.000040
Exoc5: exocyst complex component 5	1436817_at	AV025913	1.70	0.003981
B230380D07Rik: RIKEN cDNA B230380D07 gene	1436841_at	AV229336	1.84	0.040661
Arl13b: ADP-ribosylation factor-like 13B	1437021_at	AV225959	1.59	0.000559
Eif1ay: eukaryotic translation initiation factor 1A, Y-linked	1437071_at	BB471576	1.55	0.024542
Slc18a2: solute carrier family 18 (vesicular monoamine), member 2	1437079_at	AV334638	2.71	0.002010
Rnps1: ribonucleic acid binding protein S1	1437359_at	BI793607	−1.55	0.017189
Acvr2a: activin receptor IIA	1437382_at	BG066107	1.71	0.005407
Mm.138561.1	1438307_at	AV317732	1.54	0.008071
Fars2: phenylalanine-tRNA synthetase 2 (mitochondrial)	1439406_x_at	BB530332	−1.56	0.015768
Sgol1: shugoshin-like 1 (*S. pombe*)	1439510_at	BB410537	1.56	0.000354
Mm.44035.1	1440222_at	BB530180	−1.87	0.004195
Mm.33045.1	1440272_at	BB232473	1.58	0.001142
Sbno2: strawberry notch homolog 2 (*Drosophila*), mRNA (cDNA clone IMAGE:3376209)	1441840_x_at	BB533975	−2.24	0.002180
Mm.37220.1	1444785_at	AI503808	−1.72	0.011949
… Predicted gene/similar to glyceraldehyde-3-phosphate dehydrogenase (GAPDH) …	1447999_x_at	AI840508	−1.53	0.005202
Rab1: RAB1, member RAS oncogene family	1448210_at	AW108405	1.65	0.000205
Lrrfip1: leucine rich repeat (in FLII) interacting protein 1	1448487_at	NM_008515	1.60	0.002779
Pafah1b1: platelet-activating factor acetylhydrolase, isoform 1b, beta1 subunit	1448578_at	BE688382	1.66	0.001433
Siah1a: seven *in absentia* 1A	1449733_s_at	AA982064	1.66	0.006169
Kpna3: karyopherin (importin) alpha 3	1450386_at	BM213828	1.53	0.006954
Twsg1: twisted gastrulation homolog 1 (*Drosophila*)	1450388_s_at	BC004850	1.54	0.003421
Stk17b: serine/threonine kinase 17b (apoptosis-inducing)	1450997_at	AV173139	2.04	0.003338
Yipf3: Yip1 domain family, member 3	1451284_at	BC019384	−1.64	0.026951
LOC100044383 /// Pnpt1: similar to polynucleotide phosphorylase-like protein /// polyribonucleotide nucleotidyltransferase 1	1452676_a_at	BB777815	1.67	0.000248
6820431F20Rik: RIKEN cDNA 6820431F20 gene	1452997_at	BE692399	1.85	0.009694
Gas2l3: growth arrest-specific 2-like 3	1453416_at	BE199211	2.05	0.004200
Usp15: ubiquitin specific peptidase 15	1454036_a_at	AK014891	1.57	0.028362
Arfip1: ADP-ribosylation factor interacting protein 1	1454916_s_at	AV087417	1.59	0.000091
Alg10b: asparagine-linked glycosylation 10 homolog B (yeast, alpha-1,2-glucosyltransferase)	1454917_at	BB795206	1.63	0.007541
Mm.24436.1	1455206_at	BQ175276	1.51	0.014053
Ccdc127: coiled-coil domain containing 127	1455248_at	AW542786	1.71	0.000473
Map3k7: mitogen-activated protein kinase kinase kinase 7	1455441_at	AW547374	1.77	0.003661
Mm.178349.1	1456547_at	BM119402	−2.02	0.026517
Lyrm5: LYR motif containing 5 (Lyrm5), mRNA	1459793_s_at	AV301944	1.72	0.009359
Dnaja1: DnaJ (Hsp40) homolog, subfamily A, member 1	1460179_at	BF141076	1.75	0.000232
Sfrs2ip: splicing factor, arginine/serine-rich 2, interacting protein	1460445_at	AK012092	1.63	0.000533
AI848100: expressed sequence AI848100	1460573_at	BM240684	1.51	0.000521

*Transcripts that fulfilled the criteria of 1.2 and 0.05 for fold change and p-value respectively for IGF-I versus the control and versus insulin and IGF-II were selected. Transcripts also regulated by insulin or IGF-II versus the control were excluded. The transcripts were then filtered for a fold change of 1.5 in comparison to the control*.

According to IPA the top five molecular and cellular functions these transcripts are involved in are molecular transport, protein trafficking, post-translational modification, protein folding, and cell morphology.

#### Transcripts selectively regulated by IGF-II

Eight transcripts were only regulated by IGF-II (see Table 4; fold change cut-off 1.5; for fold changes and *p*-values for insulin and IGF-I: see Table S6 in Supplementary Material). Two of these transcripts were TNF receptor-associated factor 1 (Traf1) and TRAF and TNF receptor-associated protein (Ttrap), which are functionally related proteins.

**Table 4:** **Transcripts selectively regulated by IGF-II**.

Transcript	Probe set (Affymetrix)	Accession nr.	Fold change	*p*-Value
Jun oncogene	1417409_at	NM_010591	1.72	0.002886
LOC100046232 /// Nfil3: similar to NFIL3/E4BP4 transcription factor /// nuclear factor, interleukin 3, regulated	1418932_at	AY061760	1.55	0.007144
expressed sequence AI467606	1433465_a_at	BB234337	1.99	0.004292
MOB1, Mps one binder kinase activator-like 2A (yeast)	1434388_at	BB023868	1.50	0.006665
LOC632433: ADP-ribosylation factor-like 4C /// similar to ADP-ribosylation factor-like protein 7	1436512_at	BI964400	1.75	0.005263
LOC634417: fos-like antigen 2 /// similar to fos-like antigen 2	1437247_at	BM245170	1.78	0.007075
TNF receptor-associated factor 1 (Traf1), mRNA	1445452_at	BB218245	1.77	0.022057
Traf and TNF receptor-associated protein	1448706_at	NM_019551	−1.68	0.000103

*Transcripts that fulfilled the criteria of 1.2 and 0.05 for fold change and p-value respectively for IGF-II versus the control and versus insulin and IGF-I were selected. Transcripts also regulated by insulin or IGF-I versus the control were excluded. The transcripts were then filtered for a fold change of 1.5 in comparison to the control*.

#### Transcripts selectively regulated by insulin

Four transcripts were only regulated by insulin (see Table 5; fold change cut-off 1.5; for fold changes and *p*-values for IGF-I and IGF-II: see Table S7 in Supplementary Material).

**Table 5:** **Transcripts selectively regulated by insulin**.

Transcript	Probe set (Affymetrix)	Accession nr.	Fold change	*p*-Value
Solute carrier family 39 (zinc transporter), member 10	1433751_at	BM250411	−2.01	0.001528
Mm.168098.1	1444326_at	BB414484	1.55	0.030559
Kruppel-like factor 6	1447448_s_at	C86813	−2.35	0.009036
Kruppel-like factor 6	1433508_at	AV025472	−1.59	0.011606

*Transcripts that fulfilled the criteria of 1.2 and 0.05 for fold change and p-value respectively for insulin versus the control and versus IGF-I and IGF-II were selected. Transcripts also regulated by IGF-I or IGF-II versus the control were excluded. The transcripts were then filtered for a fold change of 1.5 in comparison to the control*.

### Gene regulation patterns of ligand pairs

#### Transcripts selectively or more potently regulated by the IGFs than by insulin

Sixty five transcripts fulfilled the set criteria for IGF-I and IGF-II in comparison to the control and to insulin. The IGFs regulated 46 transcripts that were not regulated by insulin in comparison to the control (Table 6). Interestingly, the 19 transcripts that were also regulated by insulin were always more regulated by the IGFs than by insulin.

**Table 6:** **Transcripts selectively or more potently regulated by the IGFs than by insulin**.

Transcript	Probe set (Affymetrix)	Accession nr.	FC IGF-I	*p*-Value IGF-I	FC IGF-II	*p*-Value IGF-II	FC insulin	*p*-Value insulin
*Dusp6: dual specificity phosphatase 6*	1415834_at	NM_026268	2.96	0.000037	4.45	0.000114	1.66	0.024401
Jun-B: Jun-B oncogene	1415899_at	NM_008416	1.97	0.000470	3.03	0.000185	1.22	0.107818
Klf10: Kruppel-like factor 10	1416029_at	NM_013692	2.28	0.006007	2.58	0.000466	1.34	0.004550
*Errfi1: ERBB receptor feedback inhibitor 1*	1416129_at	NM_133753	2.48	0.000009	3.30	0.000797	1.50	0.002918
Nfe2l2: nuclear factor, erythroid derived 2, like 2	1416543_at	NM_010902	1.77	0.000045	1.59	0.000011	1.10	0.286909
Egr1: early growth response 1	1417065_at	NM_007913	2.06	0.000005	2.51	0.000152	1.37	0.002267
*Ptgs2: prostaglandin-endoperoxide synthase 2*	1417262_at	M94967	5.01	0.002321	5.83	0.001415	1.88	0.001026
*Ptgs2: prostaglandin-endoperoxide synthase 2*	1417263_at	M94967	5.12	0.004035	5.86	0.003544	1.82	0.010523
Klf4: Kruppel-like factor 4 (gut)	1417394_at	BG069413	2.93	0.000277	2.91	0.001910	1.34	0.019087
Klf4: Kruppel-like factor 4 (gut)	1417395_at	BG069413	2.38	0.000136	2.36	0.001509	1.10	0.330387
*Ccnd1: cyclin D1*	1417420_at	NM_007631	2.19	0.000812	2.49	0.000605	1.53	0.008594
*Ddit3: DNA-damage inducible transcript 3*	1417516_at	NM_007837	3.90	0.000022	3.52	0.007483	1.95	0.003623
*Bhlhe40: basic helix-loop-helix family, member e40*	1418025_at	NM_011498	2.42	0.000026	3.46	0.000576	1.66	0.005081
Rbpj: recombination signal binding protein for immunoglobulin kappa J region	1418114_at	NM_009035	1.64	0.001503	1.64	0.042760	−1.01	0.869918
*HB-EGF: heparin-binding EGF-like growth factor*	1418349_at	L07264	2.93	0.000389	4.11	0.003861	1.67	0.016481
HB-EGF: heparin-binding EGF-like growth factor	1418350_at	L07264	2.37	0.000879	3.43	0.002878	1.39	0.003493
*Fzd2: frizzled homolog 2 (Drosophila)*	1418533_s_at	BB371406	−2.73	0.002491	−2.72	0.001879	−1.74	0.008244
Snai2: snail homolog 2 (*Drosophila*)	1418673_at	NM_011415	2.55	0.003833	2.43	0.016438	1.45	0.039762
Arc: activity regulated cytoskeletal-associated protein	1418687_at	NM_018790	3.46	0.004766	5.51	0.014618	1.72	0.065388
Phlda1: pleckstrin homology-like domain, family A, member 1	1418835_at	NM_009344	2.50	0.000016	3.42	0.000137	1.45	0.006783
*Ereg: epiregulin*	1419431_at	NM_007950	3.81	0.003989	4.98	0.007224	1.59	0.013385
Errfi1: ERBB receptor feedback inhibitor 1	1419816_s_at	AI788755	2.18	0.000303	2.82	0.003084	1.43	0.013860
*Vegfa: vascular endothelial growth factor A*	1420909_at	NM_009505	3.57	0.003003	3.60	0.001070	2.14	0.049047
*Areg: amphiregulin*	1421134_at	NM_009704	18.39	0.004443	32.85	0.001366	6.46	0.018404
Hmga2: high mobility group AT-hook 2	1422851_at	X58380	2.17	0.012765	2.90	0.015282	1.20	0.178787
Fos: FBJ osteosarcoma oncogene	1423100_at	AV026617	2.79	0.000301	3.58	0.000988	1.43	0.011280
Spred1: sprouty protein with EVH-1 domain 1, related sequence	1423160_at	BQ044290	1.65	0.002015	1.79	0.003587	1.18	0.246347
Spred1: sprouty protein with EVH-1 domain 1, related sequence	1423161_s_at	BQ044290	2.04	0.003684	1.95	0.004457	1.24	0.055176
Socs5: suppressor of cytokine signaling 5	1423350_at	AA510713	1.74	0.000238	2.15	0.001624	1.25	0.041765
Eif1a: eukaryotic translation initiation factor 1A	1424344_s_at	BM200591	2.33	0.004717	1.79	0.023539	1.12	0.358396
*Myc: myelocytomatosis oncogene*	1424942_a_at	BC006728	2.57	0.001522	3.41	0.001457	1.57	0.004404
Ppm1a: protein phosphatase 1A, magnesium dependent, alpha isoform	1425537_at	AF259672	1.91	0.021188	1.70	0.022908	1.02	0.912487
Egr2: early growth response 2	1427682_a_at	X06746	2.39	0.000571	3.21	0.001597	−1.03	0.747696
Egr2: early growth response 2	1427683_at	X06746	2.35	0.000002	3.19	0.000841	−1.16	0.214812
Cdc42ep2: CDC42 effector protein (Rho GTPase binding) 2	1428750_at	BF453885	−2.77	0.000119	−2.53	0.000292	−1.30	0.080566
Dusp4: dual specificity phosphatase 4	1428834_at	AK012530	3.66	0.005728	5.33	0.003373	1.53	0.118230
Zbtb2: zinc finger and BTB domain containing 2	1434901_at	BB484975	1.71	0.008994	1.68	0.004970	1.19	0.019503
Btaf1: BTAF1 RNA polymerase II, B-TFIID transcription factor-associated (Mot1 homolog, *S. cerevisiae*)	1435249_at	BG917504	2.28	0.001543	1.99	0.003586	1.34	0.009186
Prkg2: protein kinase, cGMP-dependent, type II	1435460_at	BB363188	2.41	0.000317	2.39	0.010622	1.26	0.091109
*Tmcc3: transmembrane and coiled-coil domains 3*	1435554_at	BB771888	2.94	0.000570	2.85	0.000256	1.80	0.009428
1810011O10Rik: RIKEN cDNA 1810011O10 gene	1435595_at	AV016374	2.14	0.001640	2.01	0.002508	1.01	0.959922
Egr3: early growth response 3	1436329_at	AV346607	3.82	0.000013	5.32	0.005082	1.23	0.105988
Marveld1: MARVEL (membrane-associating) domain containing 1	1436830_at	BB324084	−1.91	0.000054	−1.68	0.007970	−1.07	0.296806
Mex3b: mex3 homolog B (*C. elegans*)	1437152_at	BG072837	2.66	0.000721	3.02	0.018407	1.21	0.436275
Bmp2k: BMP2 inducible kinase	1437419_at	BB329439	2.35	0.003344	2.02	0.000029	1.39	0.033634
Zfp36l2: zinc finger protein 36, C3H type-like 2	1437626_at	BB031791	2.15	0.000301	2.53	0.011093	1.43	0.036717
C130039O16Rik: RIKEN cDNA C130039O16 gene	1444107_at	BB091357	1.60	0.010486	1.69	0.022667	−1.02	0.894938
Snai2: snail homolog 2 (*Drosophila*)	1447643_x_at	BB040443	3.22	0.010688	2.43	0.003249	1.48	0.071908
Pogk: pogo transposable element with KRAB domain	1447864_s_at	AV377712	2.20	0.016223	2.04	0.003467	1.31	0.014693
Myd116: myeloid differentiation primary response gene 116	1448325_at	NM_008654	2.00	0.000179	2.01	0.006734	1.24	0.070585
Jun: Jun oncogene	1448694_at	NM_010591	1.78	0.008793	1.90	0.011924	1.04	0.807786
*Atf3: activating transcription factor 3*	1449363_at	BC019946	2.88	0.001391	2.90	0.004451	1.92	0.005831
Ces1: carboxylesterase 1	1449486_at	NM_021456	−2.01	0.018317	−1.96	0.023919	−1.16	0.351288
Hmga2: high mobility group AT-hook 2	1450780_s_at	X58380	2.74	0.006298	3.29	0.010165	1.43	0.035048
Hmga2: high mobility group AT-hook 2	1450781_at	X58380	2.36	0.018209	3.22	0.007611	1.31	0.019092
*Gtpbp4: GTP binding protein 4*	1450873_at	AI987834	3.10	0.000236	2.75	0.006583	1.87	0.002062
Pvr: poliovirus receptor	1451160_s_at	BB049138	2.21	0.011238	2.13	0.002468	1.47	0.001190
Arl4c /// LOC632433: ADP-ribosylation factor-like 4C /// similar to ADP-ribosylation factor-like protein 7	1454788_at	BQ176306	1.70	0.005522	1.57	0.022003	1.00	0.976940
Zbtb11: zinc finger and BTB domain containing 11	1454826_at	BM195115	2.04	0.001240	1.78	0.015361	1.11	0.277271
*Foxn2: forkhead box N2*	1454831_at	AV221013	2.85	0.000516	2.85	0.004267	1.71	0.037944
Tmcc3: transmembrane and coiled-coil domains 3	1454889_x_at	BB711990	1.99	0.000120	1.89	0.003292	1.29	0.001608
Spty2d1: SPT2, Suppressor of Ty, domain containing 1 (*S. cerevisiae*)	1455130_at	BM242524	2.06	0.000339	2.04	0.000495	1.34	0.043771
*Plcxd2: phosphatidylinositol-specific phospholipase C, X domain containing 2*	1455324_at	BQ176176	4.03	0.000971	3.50	0.005766	2.21	0.007254
*LOC631639 /// Lonrf1: similar to CG32369-PB, isoform B /// LON peptidase N-terminal domain and ring finger 1*	1455665_at	BB705689	4.56	0.003239	4.17	0.002087	2.17	0.008852
Nfkbie: nuclear factor of kappa light polypeptide gene enhancer in B-cells inhibitor, epsilon	1458299_s_at	BB820441	1.71	0.000989	1.94	0.002873	1.24	0.053832

*Transcripts that fulfilled the criteria of 1.2 and 0.05 for fold change and p-value respectively for IGF-I and IGF-II versus the control and versus insulin were selected. The transcripts were then filtered for a fold change of 1.5 in comparison to the control. Transcripts that also fulfilled the criteria for insulin versus the control are in italic. FC, fold change*.

The top five molecular and cellular functions in IPA for these genes were cellular development, cellular growth and proliferation, cell cycle, gene expression, and cell death and survival. Two of the top five canonical pathways represented by these genes were ErbB signaling and neuregulin signaling. The regulated transcripts in these pathways were amphiregulin, epiregulin, heparin-binding EGF-like growth factor, FBJ osteosarcoma oncogene, and Jun oncogene for ErbB signaling and amphiregulin, epiregulin, heparin-binding EGF-like growth factor, ERBB receptor feedback inhibitor 1, and myelocytomatosis oncogene for neuregulin signaling.

#### Selective gene regulation by insulin and IGF-II

Twenty transcripts fulfilled the criteria for insulin and IGF-II in comparison to the control and to IGF-I (Table 7). Fourteen of these were not influenced by IGF-I in comparison to the control, while they were either down-regulated or up-regulated by insulin and IGF-II.

**Table 7:** **Selective gene regulation by insulin and IGF-II**.

Transcript	Probe set (Affymetrix)	Accession nr.	FC insulin	*p*-Value insulin	FC IGF-II	*p*-Value IGF-II	FC IGF-I	*p*-Value IGF-I
*Dusp6: dual specificity phosphatase 6*	1415834_at	NM_026268	1.66	0.024401	4.45	0.000114	2.96	0.000037
Nusap1: nucleolar and spindle associated protein 1	1416309_at	BC009096	−1.61	0.000141	−1.61	0.000022	−1.14	0.097812
Ndc80: NDC80 homolog, kinetochore complex component (*S. cerevisiae*)	1417445_at	NM_023294	−1.73	0.000121	−1.61	0.000253	−1.16	0.056240
Ghr: growth hormone receptor	1417962_s_at	NM_010284	−1.67	0.008693	−1.69	0.008944	−1.15	0.197168
*Bhlhe40: basic helix-loop-helix family, member e40*	1418025_at	NM_011498	1.66	0.005081	3.46	0.000576	2.42	0.000026
*Nfyb: nuclear transcription factor-Y beta*	1419267_at	AV250496	1.53	0.007996	1.60	0.005883	2.43	0.005169
*Areg: amphiregulin*	1421134_at	NM_009704	6.46	0.018404	32.85	0.001366	18.39	0.004443
PQlc2: PQ loop repeat containing 2	1425632_a_at	BC019216	2.31	0.001027	2.12	0.001076	1.40	0.029326
Cebpb: CCAAT/enhancer binding protein (C/EBP), beta	1427844_a_at	AB012278	1.74	0.018553	1.80	0.005586	1.13	0.445063
Sema3c: sema domain, immunoglobulin domain (Ig), short basic domain, secreted (semaphorin) 3C	1429348_at	AK004119	−1.70	0.006766	−1.72	0.008802	1.03	0.733222
Cyld: cylindromatosis (turban tumor syndrome)	1429617_at	BM119209	−1.61	0.005787	−1.50	0.003897	−1.03	0.807135
Bop1: block of proliferation 1	1430491_at	AV128350	1.78	0.013556	1.93	0.006039	1.04	0.820081
Rhobtb3: Rho-related BTB domain containing 3	1433647_s_at	BM942043	−1.62	0.027000	−1.64	0.022981	−1.02	0.890963
*Sc5d: sterol-C5-desaturase (fungal ERG3, delta-5-desaturase) homolog (S. cerevisae)*	1434520_at	AU067703	2.18	0.006626	2.25	0.001725	3.34	0.000004
Foxp1: forkhead box P1	1435222_at	BM220880	−2.10	0.010890	−1.94	0.017867	−1.44	0.055486
Kif11: kinesin family member 11	1435306_a_at	BM234447	−1.92	0.003119	−1.76	0.006149	−1.20	0.115116
Ppm2c: protein phosphatase 2C, magnesium dependent, catalytic subunit	1438201_at	AV290622	−2.18	0.000445	−1.54	0.028117	1.05	0.650024
*Matr3: Matrin 3, mRNA (cDNA clone MGC:28206 IMAGE:3989914)*	1441272_at	BI249188	2.63	0.004643	2.78	0.000614	1.72	0.006058
Kif11: kinesin family member 11	1452314_at	BB827235	−2.02	0.003923	−1.54	0.017306	1.11	0.406989
Kif11: kinesin family member 11	1452315_at	BB827235	−1.85	0.000158	−1.83	0.000706	−1.13	0.347961

*Transcripts that fulfilled the criteria of 1.2 and 0.05 for fold change and p-value respectively for insulin and IGF-II versus the control and versus IGF-I were selected. The transcripts were then filtered for a fold change of 1.5 in comparison to the control. Transcripts that also fulfilled the criteria for IGF-I versus the control are in italic. FC, fold change*.

The top five molecular and cellular functions in IPA for the 14 genes specifically regulated by insulin and IGF-II were cell cycle, cellular assembly and organization, DNA replication, recombination and repair, cellular function and maintenance, and cell morphology.

#### Gene regulation by insulin and IGF-I

Eleven transcripts fulfilled the criteria for insulin and IGF-I in comparison to the control and to IGF-II (Table 8). In contrast to the selective gene regulation by the IGFs and by insulin and IGF-II, 10 of these 11 transcripts were also, and more strongly, influenced by IGF-II.

**Table 8:** **Gene regulation by insulin and IGF-I**.

Transcript	Probe set (Affymetrix)	Accession nr.	FC insulin	*p*-Value insulin	FC IGF-I	*p*-Value IGF-I	FC IGF-II	*p*-Value IGF-II
*Dusp6: dual specificity phosphatase 6*	1415834_at	NM_026268	1.66	0.024401	2.96	0.000037	4.45	0.000114
*Slc40a1: solute carrier family 40 (iron-regulated transporter), member 1*	1417061_at	AF226613	−2.63	0.000644	−2.99	0.000973	−4.48	0.000804
*Fosl1: fos-like antigen 1*	1417487_at	U34245	3.85	0.002671	4.54	0.000291	7.87	0.003065
*Fosl1: fos-like antigen 1*	1417488_at	U34245	4.48	0.001278	5.31	0.001160	8.69	0.001387
*Bhlhe40: basic helix-loop-helix family, member e40*	1418025_at	NM_011498	1.66	0.005081	2.42	0.000026	3.46	0.000576
Rgs2: regulator of G-protein signaling 2	1419248_at	AF215668	1.69	0.003144	1.99	0.033929	1.04	0.849605
*Areg: amphiregulin*	1421134_at	NM_009704	6.46	0.018404	18.39	0.004443	32.85	0.001366
*LOC100047324 /// Sesn1: similar to Sesn1 protein /// sestrin 1*	1433711_s_at	BG076140	−1.63	0.016249	−1.71	0.017257	−2.64	0.002200
*Plk3: polo-like kinase 3 (Drosophila)*	1434496_at	BM947855	2.74	0.002507	2.21	0.007719	4.79	0.000021
*Mm.52043.1*	1437199_at	BB442784	2.05	0.035779	2.27	0.022600	4.81	0.000445
*D8Ertd82e: DNA segment, Chr 8, ERATO Doi 82, expressed*	1442434_at	BM195829	2.17	0.008597	2.55	0.004759	4.47	0.001872

*Transcripts that fulfilled the criteria of 1.2 and 0.05 for fold change and p-value respectively for insulin and IGF-I versus the control and versus IGF-II were selected. The transcripts were then filtered for a fold change of 1.5 in comparison to the control. Transcripts that also fulfilled the criteria for IGF-II versus the control are in italic. FC, fold change*.

### Validation of the microarray data by qRT-PCR

**Figure 2:**
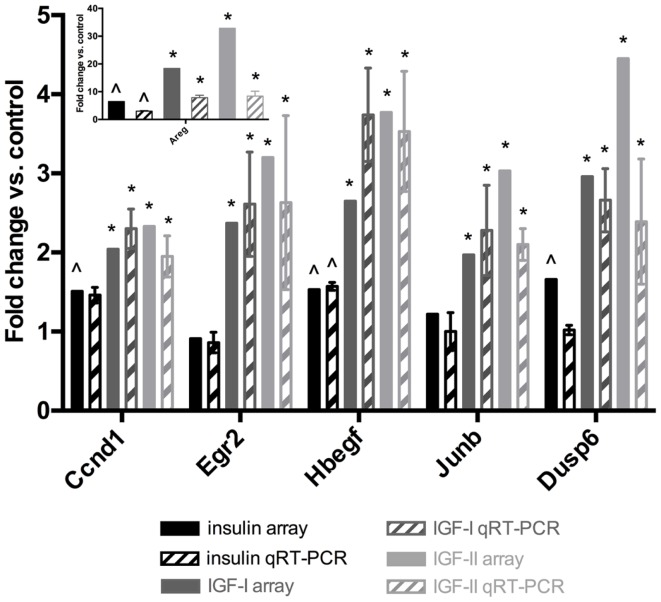
**Validation of microarray data by qRT-PCR**. Two-step RT-PCR was performed on a subset of transcripts. 18S was used as an internal control. The results are expressed as fold change in comparison to the control (unstimulated samples). Full bars represent the microarray data (Table 6). Bars with patterns represent the average qRT-PCR results ± standard deviations. Black: insulin, gray: IGF-I, light gray: IGF-II. Significant differences in the qRT-PCR data were calculated by a two-tailed *t*-test. ^∧^Significantly up-regulated by insulin in comparison to the control at the 1.5 fold change and 0.05 *p*-value level. *Significantly more up-regulated by this IGF than by insulin at the 0.05 *p*-value level.

To validate the microarray data qRT-PCR was performed for six transcripts on the total RNA of three independent biological replicates. These RNA samples are independent of the RNA used to generate the microarray data. Fold changes were calculated in comparison to the control and plotted in Figure 2. For the IGFs, the regulation trends from the microarray experiments (Table 6) are confirmed by qRT-PCR for all six genes: the IGFs regulate these genes more potently than insulin. For insulin, gene regulation (Table 6) was confirmed for four out of six genes (Areg, Egr2, HB-EGF, and Jun-B). In addition, for Ccnd1 the fold change was 1.51 on the array and 1.46 by qRT-PCR, two values that lay very close and are only just separated by the 1.5 fold change cut-off. In conclusion, the qRT-PCR data validate very well the microarray results.

## Discussion

We compared the gene expression responses stimulated by insulin, IGF-I, and IGF-II through the IGF-I receptor using Affymetrix gene expression profiling. In order to eliminate the influence of the affinity of the ligands stimulating the receptor, we stimulated the IGF-I receptor on a mouse fibroblast cell line with concentrations of insulin, IGF-I, and IGF-II that compensated for the relative affinities of the ligands for the receptor on this cell line. Our analyses revealed that these three ligands stimulate overlapping but specific gene expression responses.

Some of the regulated transcripts that appeared in our analyses were also found by Mulligan et al. who studied the gene expression pattern after stimulating a chimeric receptor containing the intracellular domain of the IGF-I receptor (30), and Dupont et al. who studied gene expression after stimulation of the IGF-I receptor with IGF-I (31). As in our study, Mulligan et al., e.g., found the up-regulation of heparin-binding EGF-like growth factor and Dupont et al. found the up-regulation of early growth response 1 and Jun oncogene. The fact that transcripts regulated after stimulation of the IGF-I receptor with IGF-I found in our study and, e.g., the one by Dupont et al. only partially overlap, is most likely due to the differences in experimental set-up. We used a different cell line, concentrations of ligands, stimulation time, microarray platform and normalization, and analysis methods and criteria.

Boucher et al. recently showed that IGF-I and insulin, at equal concentrations, regulate the expression of the same genes through the IGF-I receptor (15). Insulin does that with a smaller magnitude of response than IGF-I. We show here that when compensating for the different affinities of the ligands, each ligand does specifically influence the expression of certain genes through the IGF-I receptor.

Each ligand specifically regulated a group of transcripts that was not regulated by the other two ligands. When stimulating the IGF-I receptor with IGF-II for example, two of the eight specifically regulated genes were Traf1 and Ttrap. Traf1 was up-regulated by IGF-II and is an inhibitor of apoptosis, which may be due to increased activation of nuclear factor-kappa B (NF-κB), an anti-apoptotic transcription factor (32– 34). Ttrap was down-regulated by IGF-II and inhibits the transcriptional activation of NF-κB (35). These results are consistent with the known anti-apoptotic activity of IGF-II through the IGF-I receptor.

In order to identify common gene regulation patterns between ligands, we studied the gene expression induced by two ligands in comparison to the control and to the third ligand. Interestingly, a group of 65 transcripts was identified to be selectively or more potently regulated by the IGFs than by insulin. ErbB signaling and neuregulin signaling were significant canonical pathways over-represented in the data set; regulated transcripts in common between the two pathways were amphiregulin, epiregulin, and heparin-binding EGF-like growth factor (HB-EGF). These were up-regulated more potently by the IGFs than by insulin. Pandini et al. showed that amphiregulin, HB-EGF, and epiregulin were similarly up-regulated by insulin and IGF-II through the insulin receptor isoform A in mouse fibroblasts (19). Mulligan et al. showed that HB-EGF transcript expression was up-regulated more potently after signaling through the IGF-I receptor than through the insulin receptor in fibroblasts (30). Amphiregulin, HB-EGF, and epiregulin are all EGF receptor (also named ErbB-1 or HER1) ligands (36). HB-EGF acts both as a regulated autocrine/paracrine and a juxtacrine growth factor (36, 37). Amphiregulin has been suggested to have both growth inhibitory and stimulatory effects (38). Epiregulin is a growth promoter in primary rat hepatocytes (39, 40) and an autocrine growth factor in human keratinocytes (41). HB-EGF and amphiregulin also bind and activate ErbB-3 and HB-EGF binds and activates ErbB-4 (42), just like the neuregulins, which bind ErbB-3 and ErbB-4. HB-EGF induces chemotaxis after stimulation of ErbB-4 (43).

As for the IGFs, we identified 14 transcripts selectively regulated by insulin and IGF-II. Using the same analysis criteria, this was however not the case when looking at insulin and IGF-I as a group. Ten of the 11 transcripts that were regulated by insulin and IGF-I in comparison to the control and IGF-II were also regulated by IGF-II. So the IGFs on one hand and insulin and IGF-II on the other hand seem to provoke more similar gene expression patterns than insulin and IGF-I. This is in accordance with the numbers presented in Table 2. Of all the transcripts regulated by insulin in comparison to the control, a larger fraction was also regulated by IGF-II than by IGF-I, even though IGF-I overall regulated more transcripts than IGF-II.

Although some of the transcripts identified in this study were involved in metabolic functions, the overall biological patterns were of a non-metabolic nature. This is not surprising, considering the tissue origin of the cell line used. From this study, no general conclusions could thus be drawn on whether certain ligands created a more metabolic or mitogenic response in comparison to the other ligands.

Many of the functions, pathways, and genes mentioned above are consistent with the known effects of insulin, IGF-I, and IGF-II. One could thus speculate that these differences in gene expression might account for some of the different biological effects of these three ligands. It should be mentioned that these gene expression patterns were measured after stimulating the receptor with supraphysiological concentrations of ligands. Therefore studying the concentration dependence of these gene expression profiles, together with performing time series of gene expression, could provide a more subtle picture.

Since the influences of affinity of the three ligands were largely accounted for in this study, it is likely that the differences in gene expression are due to intrinsic properties of each ligand. Different suggestions have been made to explain the mechanism responsible for this signaling specificity. Both differences in ligand binding kinetics and internalization properties have been correlated with different responses after stimulating the insulin receptor with different ligands (21–23, 44–46). More studies are needed in order to clarify at which level the cellular signal of different ligands stimulating the same receptor diverges.

## Conclusion

We studied the gene expression patterns after stimulating the IGF-I receptor with equipotent concentrations of IGF-I, IGF-II, and insulin by microarray gene expression profiling and found significant differences in responses between the three ligands. Each ligand specifically regulated a group of transcripts that was not regulated by the other two ligands. Also, insulin and IGF-I seemed to stimulate the least overlapping response. The different gene expression profiles for the three ligands might explain some of their different biological effects. These results also add to the accumulating evidence that different ligands can bind to the same receptor and stimulate different cellular responses and that the nature of a ligand bound to a receptor, and not just its concentration and affinity, is determinant for the downstream cellular response. Further studies should help bringing a mechanistic understanding to the different functional consequences of different ligands activating the same receptor.

## Conflict of Interest Statement

The authors declare that the research was conducted in the absence of any commercial or financial relationships that could be construed as a potential conflict of interest.

## Supplementary Material

The Supplementary Material for this article can be found online at http://www.frontiersin.org/Molecular_and_Structural_Endocrinology/10.3389/fendo.2013.00098/abstract

Supplementary Table S1 **Transcripts regulated by insulin and the IGFs**.

Supplementary Table S2 **Transcripts regulated by IGF-I**.

Supplementary Table S3 **Transcripts regulated by IGF-II**.

Supplementary Table S4 **Transcripts regulated by insulin**.

Supplementary Table S5 **Transcripts selectively regulated by IGF-I**.

Supplementary Table S6 **Transcripts selectively regulated by IGF-II**.

Supplementary Table S7 **Transcripts selectively regulated by insulin**.

## Footnotes

^1^http://www.r-project.org/

^2^http://www.bioconductor.org/

^3^http://www.dchip.org

^4^http://www.ingenuity.com
